# Ablation of advanced tongue cancer and mobile tongue reconstruction by using a sensitive anterolateral thigh and vastus lateralis muscle free flap

**Published:** 2015

**Authors:** Tuhar, D Zamfirescu, C Gheorghiță, D Slăvescu, A Frunză, I Lascăr

**Affiliations:** * Clinic of Plastic Surgery and Reconstructive Microsurgery, “Carol Davila” Military Emergency Hospital, Bucharest, Romania; ** Clinic of Plastic Surgery and Reconstructive Microsurgery, Bucharest Emergency Hospital, Bucharest, Romania; *** “Carol Davila” University of Medicine and Pharmacy, Bucharest, Romania

**Keywords:** free flap, anterolateral thigh flap, tongue reconstruction

## Abstract

**Background.** Successful tongue reconstruction after total glossectomy for advanced tongue or base of tongue cancer should restore swallowing, speech function, and cosmesis.

**Methods.** The anterior lateral thigh flap sensitive myocutaneous (ALTF) with vastus lateralis muscle was used to reconstruct the oral defect in a patient undergoing total glossectomy with laryngeal preservation for T4 tongue cancer.

**Results.** Good functional outcomes, measured by independent feeding, speech and swallowing, were achieved.

**Conclusions.** The anterolateral thigh myocutaneous flap for total tongue reconstruction creates a free neotongue tip with an adequate volume, producing acceptable swallowing function and cosmesis. The reconstruction with free flaps is a feasible method of restoring the functional outcomes in speech and deglutition among patients who undergo total glossectomy with laryngeal preservation.

## Introduction

Extensive defects of the tongue, which result after tumor resections, represent a difficult problem for the plastic surgeon, especially if he tries to cover the defect in a single operator time and if he wants the resection to be made in the healthy tissue in order to avoid local recurrence [**1**-**5**]. Free flaps allow the one-stage reconstruction, and this is the main reason why the most complicated technique has become very used in head and neck surgery. For patients with resection and free flap reconstruction, the oral tongue or the base of the tongue, significant decline in speech and swallowing function develop in the early postoperative phase, but the patient recovers after 1 year. The reconstruction of the large carcinologic tongue defect is mandatory to recover adequate speech and swallowing. Free flaps provide thin and pliable tissues needed to restore the shape and the volume of the tongue but their functional outcomes, especially in the case of total mobile tongue reconstruction, are still limited.

Antero-lateral thigh flap was firstly described in 1983 by Baekin, followed shortly afterwards by Song et al. in 1984. This flap has several advantages, but it is still not widely used due to the different anatomic ways, thus localization of the perforators being hardly estimated before operation. There are studies that prove the utility of color Doppler ultrasound, which can accurately establish the localization of the perforators, thus influencing the planning of the antero-lateral thigh flap. Flap sizes vary from 11/ 5 to 26/ 8 cm. If a bigger flap is taken, the donor site must be grafted. The flap can be taken as a sensitive flap on the lateral femoral cutaneous nerve. Vascularisation of the flap is ensured by the descendent branch of the lateral circumflex femoral artery, with a diameter between 1,5 and 2,5 mm and a length of approximately 7 cm. The venous drainage is ensured by the two comittant veins which have a diameter slightly larger than the artery’s [**6**-**10**].

The tegument and the subcutaneous cellular tissue of the antero-lateral thigh area in some patients is thin and pliable, this area having the possibility of being a donor area for a large, pliable, thin and sensitive fasciocutaneous flap (if the defect needs such reconstructions). The cutaneous island can reach 25/ 8 cm and it can be closed by direct suture. The flap has a large vascular pedicle but there is a quite big anatomic variability. Most of the flaps need a dissection of the musculocutaneous perforators, these being variably supplied by septocutaneous perforators. It is a flap difficult to dissect, thus it is not advisable for the surgeons with less experience to lift such flaps [**11**].

The pattern of the anterolateral thigh flap is placed on the septum between the vast lateral muscle and the right femoral muscle. Arterial vascularization is given by the descendent branch of the lateral circumflex femoral artery, which has its origin in the deep femoral artery. Cutaneous branches vascularize the skin situated superficially from the vast lateral muscle [**12**,**13**]. The pedicle can be of 7-8 cm long and the vascular diameter varies between 1,5-2 mm. Usually, the artery has 2 comittant veins with a diameter larger than the artery. The flap can be enervated by the main branches of the lateral femoral cutaneous nerve (L2-3). This branch enters the flap in the upper part and it can be dissected proximally to gain length. It has a tract along the flap axis, from the anterior superior iliac spine (SIAS) to the lateral edge of the knee [**14**,**15**].

The closing of the donor area can be made by direct suture. If the defect is large, a skin graft is applied at the donor site [**17**].

## Case Presentation

Patient, male, aged 61, non-smoker, hospitalized with a diagnosis of lingual tumor with recent history, with invasion on sublingual salivary gland, invasion at mandible level, oral floor, and bilateral cervical adenopathy.

Intraoperatively, the following were performed: bilateral cervical ganglion removal, total resection of the tongue, oral floor, suprahyoid musculature, mandible access osteotomy. After the tumor ablation and neck dissection made by a maxillofacial surgeon, the anterolateral thigh and vastus lateralis flap was raised by plastic surgeons and transferred into the defect, where the microvascular anastomosis was done. Microvascular artery anastomoses were performed by termino-terminal vascular suture between the lateral circumflex femoral artery and the facial artery. For the venous anastomoses, the chosen vascular pedicles were the external jugular vein and the thyroid-lingual-facial venous trunk. Flap innervations were made by motor branch neuroraphy with left hypoglossal nerve and sensitive branch with the left lingual nerve. The mandible was reconstructed by osteosynthesis with plate and screws. The donor site was closed primarily.

**Fig. 1 a,b:**
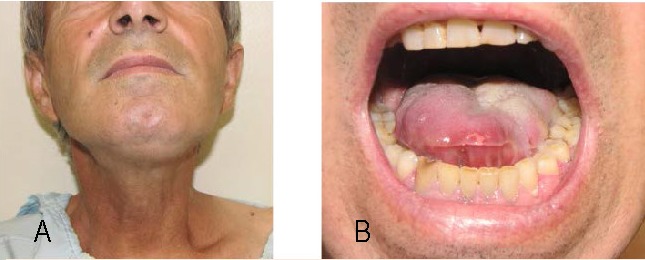
Preoperatory aspect

**Fig. 2 a,b:**
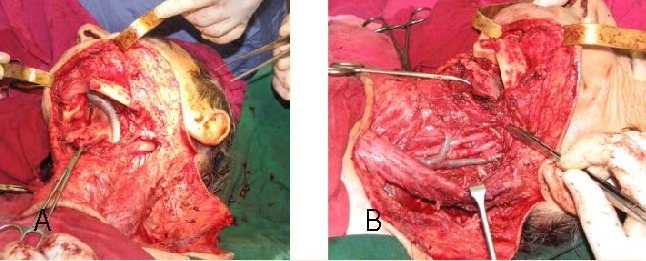
Intraoperative view

**Fig. 3:**
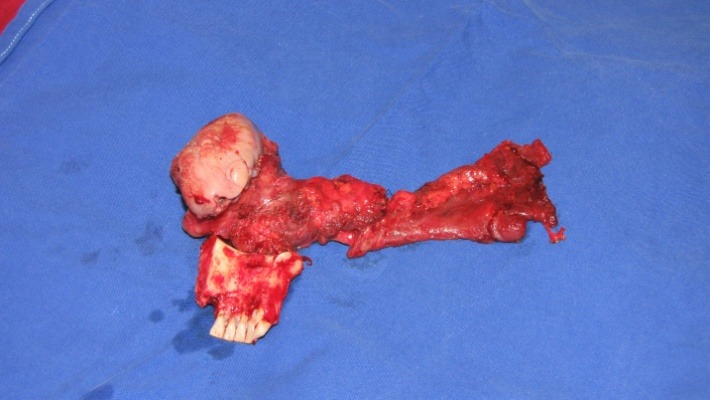
Tumor aspect

**Fig. 4 a,b:**
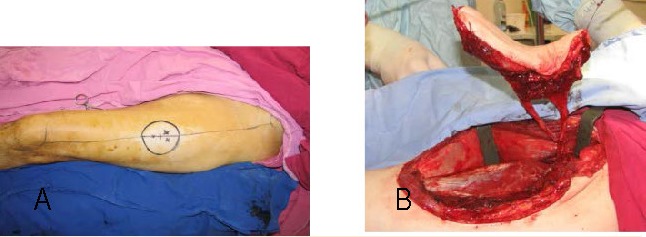
The raised flap

**Fig. 5 a,b:**
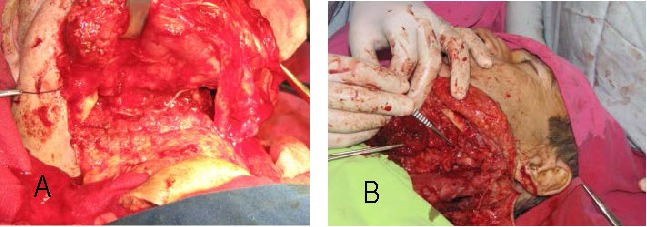
Intraoperative view

**Fig. 6 a,b:**
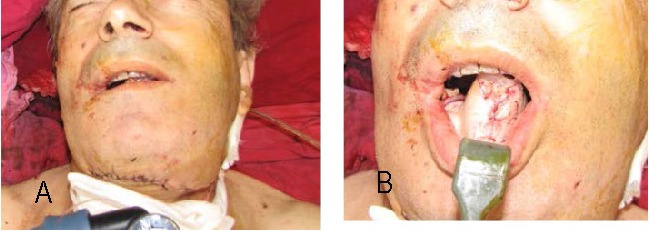
Final intraoperative aspect

**Fig. 7 a,b:**
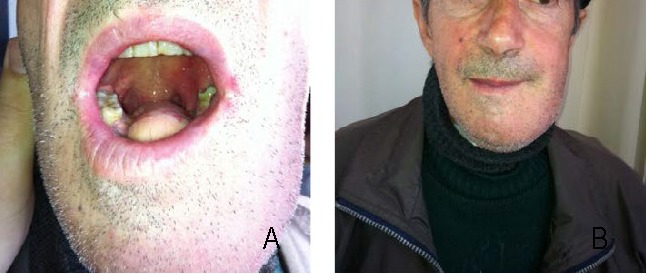
Final postoperatory aspect

## Results

The patient underwent mobile total tongue reconstruction with a sensitive anterolateral thigh that recreated a near normal neotongue shape with more projected tip and vertical bulk. This modification of the flap shaping and folding optimized the postoperative oral function.

The flap survived with no major additional complications. The patients with pelvilingual tumor needed preoperatory tracheotomies and were postoperatorily fed by a nasogastric probe. The donor site was closed primarily.

The aesthetic postoperatory aspect was good, the patient asked for flap revision aesthetically. No complications at the donor area level were presented.

## Discussions

Extensive tongue defects represent a difficult reconstructive problem for a plastic surgeon due to the affection of this complex structure [**2**,**16**]. Different flaps have been used in the reconstruction of these defects: right abdominal muscle flap, musculocutaneous latissimus dorsi flap, antebrachial radial flap, etc.

The right muscular abdominal flap is usually used for filling some deep defects at the face level [**17**]. The anterolateral thigh flap represents a very good choice for the reconstruction of the complex defects at the face level. The flap can replace large volumes of tissues and the skin island is large and can be used both for the tegument reconstruction and for the oral mucosa reconstruction. Morbidity of the donor area is minimum, thus, the direct suture is possible in all cases if the tegument island does not exceed 8 cm in width. The vascular pedicle is usually long and does not present any atheromas, the length of the pedicle can reach considerable dimensions (7-12 cm) by proximal dissection, thus being an adequate pedicle for anastomoses in the cervical region. The vascular diameter of 2-2,5 mm is joined by 2 comittant veins with the diameter of 1,8-3 mm. The drawing of the flap can consist of two tegument islands: one for the reconstruction of the mucosa and one for covering the skin, and a segment of vast lateral muscle, which ensures the closing of the dead spaces, determining an acceptable volume at the zygomatic region, ensuring the acceptable support of the orbital content by fascia fixation on the orbital periosteum. This flap can be reinnervated, which is very important in the intra-oral reconstruction, by including the lateral cutaneous femoral nerve in the flap. Post-operatory radiotherapy does not represent a contraindication and does not determine a negative effect on the flap both for a long time and for a short time follow-up.

The flap has many advantages. At dimensions that do not exceed 8 cm in width, the donor area can close primarily, damage being minimum at the level of the donor area. Two operatory teams can work simultaneously, the flap collection can be done in the same time with the ablative surgery, thus, the intervention time can be shortened. It is a flap of large dimensions, the tegument island can reach dimensions of 18/ 25 cm, ½ of the lateral thigh can be included in this flap. The anterolateral thigh free flap has become very popular in the reconstruction of the very large and complex defects of the face after cancer ablation. Despite its difficulty in the dissection and the variability of the vascular anatomy, this flap has very good results in face reconstruction and the donor site morbidity is very low.

## Discussions

The patient underwent mobile total tongue reconstruction with a sensitive anterolateral thigh that recreates a near normal neotongue shape with more projected tip and vertical bulk. This modification of the flap shaping and folding optimizes postoperative oral function. The anterolateral thigh myocutaneous flap creates a free neotongue tip with adequate volume, producing acceptable swallowing function and cosmesis.

The reconstruction with free flaps is a feasible method to restore the functional outcomes in speech and deglutition among patients who undergo total glossectomy with laryngeal preservation.

The anterolateral thigh flap ensures an acceptable post-operatory result, both from the functional and aesthetic point of view, in case of its use in covering the complex postablational defects of the tongue. The use of free flaps in the immediate reconstruction of the tongue after tumor resection should aim at the maintenance of the mobility of the residual tongue and restoration of tongue bulk in order to optimize the recovery of speech and swallowing function. The volume, shape and size of the flap can be adjusted depending on the defect. It has the same range of success as the classical free.
